# Interactions between microglia and glioma in tumor microenvironment

**DOI:** 10.3389/fonc.2023.1236268

**Published:** 2023-08-28

**Authors:** Jin-Cheng Tao, Dong Yu, Wei Shao, Dong-Rui Zhou, Yu Wang, Shi-Qiang Hou, Ke Deng, Ning Lin

**Affiliations:** ^1^ Department of Neurosurgery, The First Affiliated Hospital of Nanjing Medical University, Nanjing, Jiangsu, China; ^2^ Department of Neurosurgery, The Affiliated Chuzhou Hospital of Anhui Medical University, The First People’s Hospital of Chuzhou, Chuzhou, Anhui, China; ^3^ Shanghai Jiao Tong University School of Medicine, Shanghai, China

**Keywords:** interaction, microglia¸ glioma, microenvironment, heterogeneity, and immunotherapy

## Abstract

Gliomas, the most prevalent primary tumors in the central nervous system, are marked by their immunosuppressive properties and consequent poor patient prognosis. Current evidence emphasizes the pivotal role of the tumor microenvironment in the progression of gliomas, largely attributed to tumor-associated macrophages (brain-resident microglia and bone marrow-derived macrophages) that create a tumor microenvironment conducive to the growth and invasion of tumor cells. Yet, distinguishing between these two cell subgroups remains a challenge. Thus, our review starts by analyzing the heterogeneity between these two cell subsets, then places emphasis on elucidating the complex interactions between microglia and glioma cells. Finally, we conclude with a summary of current attempts at immunotherapy that target microglia. However, given that independent research on microglia is still in its initial stages and has many shortcomings at the present time, we express our related concerns and hope that further research will be carried out to address these issues in the future.

## 1 Introduction

The characteristics of brain tumors include high incidence and mortality rates and are usually locally aggressive in growth. Brain tumors are predominantly caused by cancers originating outside the central nervous system (CNS) that have metastasized, while gliomas and meningiomas represent the most frequently occurring primary brain tumor varieties, with gliomas constitute nearly 30% of all primary brain tumors and 80% of all malignancies. Consequently, gliomas are the primary cause of death in patients with primary brain tumors (1). Based on the World Health Organization (WHO) taxonomy, gliomas are classified into four grades based on the presence or absence of mesenchymal features, with WHO grade I indicating the lowest malignancy and grade IV the highest. Generally, grade I/II gliomas are low-grade gliomas (LGG), and grade III/IV gliomas are high-grade gliomas (HGG), which include the most malignant glioblastoma (1, 2). In general, tumor grade is negatively correlated with patient prognosis, with median survival time remaining less than 12 years even for LGG (3). Thus, the prognosis for HGG patients is worse, with a median overall survival (OS) time of approximately 3 years for grade III glioma patients, while the median OS time for grade IV glioma patients is just slightly over a year (4). However, recent discoveries indicate that patients with gliomas carrying isocitrate dehydrogenase (IDH) mutations exhibit a comparatively high survival rate compared to wild-type patients (5). Annually, around 100,000 individuals globally receive a diagnosis of diffuse glioma, but account for less than 5% of new cancer diagnoses, yet possess a high mortality rate. Of these, glioblastoma, a grade IV glioma, is the most lethal and constitutes 70-75% of all diffuse gliomas. Globally, there are significant differences in glioma incidence between Europe and Asia, with age, gender, ethnicity and tumor histology also contributing, in addition to geographically-induced differences in incidence. In addition, the age and gender of the patient also shape the final survival rate (6). The current conventional treatment for adult patients with malignant glioma is a combination of surgical resection, postoperative radiotherapy and chemotherapy. However, this standard treatment regimen has strong limitations in terms of the clinical outcomes it brings to patients. And high-grade gliomas tend to recur, which often prevents complete clinical cure of gliomas (7). At the same time, glioma patients undergoing conventional surgical treatment often cause an increased degree of loss of their own neurological function or the development of new neurological impairment, that is typically linked to an unfavorable ultimate prognosis for the patient (8).

The brain was previously considered an “immune privileged” organ with immunosuppressive properties due to its protection from various immune cells by the blood-brain barrier (BBB) (9). However, as early as 1925, Wilder Penfield first described microglia in the context of glioma and proposed that microglia had a significant function in extracellular matrix (ECM) remodeling (10). And a later research by Bettinger’s team in 2002 suggested that the remodeling effect of microglia on the ECM was pro-tumor, as the migratory capacity of glioma cells was enhanced threefold in the background of the presence of microglia (11). And in a subsequent study, microglia and blood/bone marrow macrophages (BMDMs) were identified as the predominant infiltrating cell population in gliomas, and it was confirmed that microglia and exogenous macrophages could serve as a crucial factor in the immune escape of gliomas (12, 13). Furthermore, it is proposed that gliomas are complex tumors composed of tumor and non-tumor cells, where most of the non-tumor cells are tumor-associated macrophages (TAMs) composed of microglia and exogenous macrophages, and TAMs can account for 30%-40% of the tumor volume, surpassing the quantity of infiltrating lymphocytes within the tumor by a large margin. Thus they become an important component of the tumor microenvironment (TME), providing a tumor-promoting matrix for tumor cell proliferation and invasion. Immune cells and non-tumor cells in the TME create an immunosuppressive microenvironment for glioma via the secretion of multiple cytokines, chemokines, growth factors, and other mediators that contribute substantially to glioma progression (14–16). The TME re-educates TAMs to have different gene expression profiles as well as altered functions, so that there is functional, spatial and temporal heterogeneity of action of TAMs in microglia and blood/bone marrow macrophages (BMDMs) (17).

Therefore, we can conclude that TAMs serve as a crucial factor in the TME and contribute to tumor progression through a close interplay relationship with glioma cells. However, the differences between microglia and BMDMs cell subpopulations and the mechanisms of interactions between microglia subpopulations and gliomas are not clear at present. And based on the poor prognosis of glioma under current conventional treatment, we may appropriately explore new options for microglia-based tumor treatment.

## 2 Microglia and BMDMs heterogeneity

### 2.1 Cell origin

Since the discovery of microglia in the brain by the Spanish neuroscientist Pío del Río Hortega in 1919, there has been a debate about their biological origin to date. The argument for an extracerebral origin was made when some researchers found specific macrophage antigens on the surface of microglia (18). Research on the origin of microglia has never ceased in recent years. In early transplantation experiments with green fluorescent protein (GFP)-labeled bone marrow (BM) hematopoietic cells, GFP-labeled microglia were detected in the cerebellum, striatum, and hippocampus, indicating that peripheral cells may partially replace microglia (19). Later, the Hickey WF team used a model of BM cell transplantation and symbiosis to somewhat answer the question of how microglia maintain themselves in the adult brain. The results showed that BM replaced only perivascular macrophages, but not branched ramified microglia in the brain parenchyma (20). In contrast, the study by Mildner A et al. proposed that the heterogeneitybetween microglia and BMDMs does not stem from prolonged developmental isolation but instead arises from the CNS’s local milieu, and suggested that microglia are transformed from Ly-6C^hi^CCR2^+^monocytes as precursor cells. Surprisingly, under protection of the head from radiation, any significant infiltration of cells of bone marrow origin was not observed in the brain, suggesting that this sporadic substitution was due to cell entry resulting from radiation disruption of the BBB with enhanced permeability (21). Recently, a study has used a new non-cleared marrow transplantation (NMT) murine model that precisely differentiate glioma microglia and BMDMs, and from which the two can be distinguished. This model achieves significant levels of peripheral hematopoietic chimerism without BBB disruption or cellular infiltration before tumor implantation when low-dose busulfan is administered prior to bone marrow transplantation (22). In addition, in mouse models of multiple sclerosis and autoimmune encephalitis (EAE), the two cell subpopulations of microglia andexogenous macrophages were indistinguishable from each other and they were recruited from proliferating resident precursors and blood-derived progenitor cells, respectively. Symbiosis and myeloablation was used to replace circulating progenitor cells without affecting CNS-resident microglia, but recruited monocytes disappeared, thus demonstrating that exogenous monocytes don’t ultimately contribute to the resident microglia pool (23).

Previous studies have used several of the specific experimental models described above, including bone marrow (BM) transplantation of syngeneic chimeric mice and sorting models by cell surface specific antigens. It has often been concluded that microglia will be partially replaced by exogenous monocytes or supplemented by blood-derived microglia progenitors. However, when it comes to microglia activation models as well as immune encephalitis models, it was concluded that the complementation or replacement of resident microglia pools by exogenous monocytes was not evident. Until recently fate-mapping studieshave revealed that immature yolk sac progenitor cells are the predominant initial source of microglia in the brain. The survival of the cell subpopulation is enabled by a long life span and a limited number of self-renewals (14, 24). The discovery of an ancestral marker, nestin, confirmed that microglia progenitor cells are not bone marrow hematopoietic cells. Multiple experiments above have demonstrated that microglia are resident in the CNS, but BMDMs is not a significant contributor to the microglia pool and recruited BMDMs do not persist in the CNS (24). However, recently Peng B’s team overturned the previous conclusion that repopulating microglia originated from Nestin-positive precursor cell differentiation by multiple genealogical tracing techniques, as well as the fact that no repopulating microglia of blood origin were found in any of the studies, thus also ruling out the hypothesis of blood origin of repopulating microglia and finally finding that their repopulating microglia originated exclusively from residual microglial cell proliferation (25).

Regardless of the findings of subsequent studies, we can easily conclude that microglia and BMDMs are two separate subpopulations of bone marrow cells, and although there is a small amount of substitution or complementation in the context of blood-brain barrier disruption, the two cell subpopulations should still correspond to different progenitor cells in terms of cellular origin, with obvious origin Heterogeneity of origin.We summarize in **Table 1** the heterogeneity that exists between microglia and BMDMs, including the cell origin.

**Table 1:** Microglia and BMDMs Heterogeneity.

Themes of Heterogeneity	Microglia	BMDMs	Reference
**Cell Origin**	Immature yolk sac progenitor cells and Ly-6C^hi^CCR2^+^ monocytes	Blood/bone marrow-derived monocytes	(14, 21)
**Percentage of cell subpopulations**	13%-34%	4.2%-12%	(26)
**Spatial Distribution**	Exterior and interior of the tumor	Interior of the tumor	(27)
**Temporal Distribution**	Dominant in primary diagnosis of tumors and IDH-mut	Dominant in recurrent tumors and BrM	(28–30)
**Specific Marker**	TMEM119, Sall1, P2RY12, CD11b(+)/CD45^low^, CX3CR1(+)/CCR2 (–)	CD45, CD49D, CD163, CD11b(+)/CD45^high^, CXCR1(+/-)/CCR2(+)	(26, 31–43)
**M1/2 polarized phenotype**	Inaccuracy, not applicable	Applicable	(30, 44–49)

IDH-mut, isocitrate dehydrogenase mutant; BrM,brain metastases.

### 2.2 Spatial and temporal distribution

Normally, the quantity of macrophages within the CNS is considerably lower than that of microglia and is mainly concentrated around the perivascular space, meninges, and organs adjacent to the ventricles and choroid plexus, and is seldom observed within the brain parenchyma (50). In the context of glioma, microglia constitute between 13% and 34% of the cellular population within the tumor, are distributed throughout the CNS, and are present in the tumor, the tumor periphery, and the contralateral tumor-free hemisphere. In contrast, macrophages account for a lower proportion of only 4.2-12% in the tumor and peritumor and only about 1% in the contralateral tumor-free hemisphere (26). The genetic lineage tracing approach also confirmed that the spatial and temporal distribution of microglia and BMDMs differed significantly in the tumor, with BMDMs are generally restricted to the interior of the tumor, however microglia were present both inside and outside the tumor, and both were generally enriched near the associated blood vessels inside the tumor, with BMDMs being more enriched than microglia. Microglia-derived TAMs predominate in tumors that have just been diagnosed, yet gradually decrease until they disappear as the glioma progresses. However, in the context of tumor recurrence, especially in the hypoxic tumor environment, the number of monocyte-derived TAMs exceeds this number. In contrast, in the context of BMDMs depletion, the proportion of microglia increases instead, suggesting that to some extent microglia may be regulated by the number of BMDMs occupied (27, 28). Friebel E’s team monitored resident and invasive leukocyte populations in the CNS in the context of glioma by multiple techniques of single cell proteomic analysis, immunofluorescence imaging and gene fate mapping. The results showed that microglia-derived TAMs dominate and are diffusely dispersed throughout the glioma, but they are restricted to the boundary area of the tumor and are not found in the central region of BrM (brain metastases), whereas invading exogenous macrophages dominate the immune microenvironment of BrM (29). The proliferation of both tumor-associated microglia and BMDMs showed increased proliferation in IDH-wt (wild-type) gliomas and BrM, whereas only microglia proliferation was upregulated in IDH-mut (mutant) gliomas. Simultaneously, the microglia to BMDMs ratio varied across different high-grade glioma subtypes (30). This suggests that the type of brain tumor determines the type of subject cell population infiltrating the tumor microenvironment as well as its distribution. Furthermore, in glioma specimens obtained from human patients, immune cells isolated from tumor margins demonstrated elevated expression of genes encoding cytokines (CCL3/4 and TNF) and pro-inflammatory interleukins (IL-1A, IL-1B and IL6-R), whereas immune cells isolated from tumor cores upregulated genes involved in angiogenesis (VEGFA/B) and those encoding pro-inflammatory inhibitors of cytokines (IL1RN). This phenomenon suggesting that TAMs infiltrating the tumor are predominantly pro-tumor phenotypes and also showing that microglia and BMDMs may play relatively different roles in tumor progression (51).

### 2.3 Specific marker

In addition to studies on the origin of the two cell subpopulations and their distribution in the brain, the phenotypic characteristics or markers of microglia have been a subject of heated discussion. Over an extended period, the main method to differentiate between exogenous monocytes and resident microgliain the brain was the CD45 antibody, which is generally considered to be less expressed in microglia (26). It is on the basis of this traditional method of differentiation that it has been suggested that the core cell population in gliomas is exogenous macrophages rather than microglia, as its major cell population was detected to be highly expressing CD45 (52). To explore the unique physiological role of microglia in the tumor microenvironment (TME), early studies have largely relied on the surface protein CD45 as well as cell morphology to differentiate, however, these two features are not specific markers of microglia. In addition, Badie B et al. suggested that microglia in the TME may also upregulate their own CD45 expression, reducing the accuracy of this CD45-based differentiation method (26). This is confirmed by the results of Müller A’s team, who used a new model of head-protected irradiation (HPI), which effectively avoids the effect of total body irradiation (TBI) on the BBB causing massive mononuclear cell infiltration and improves the accuracy of probing for tumor-associated cells. It is proposed that microglia in the context of glioma can increase their own CD45 expression and become part of the fixed expression of CD45 in glioma. This further reveals the obvious inadequacy of previous conventional differentiation methods (31).

At present, many experiments have used new technologies to explore the specific characterization of microglia in order to distinguish them from BMDMs cell subpopulations. Based on flow sorting techniques and transcriptome sequencing, marker molecules specific to primary microglia that are only expressed in their own were identified. Transcription factors, including Cebpe, Rhox5, E2f6, Phf17, Ppargc1b and Hoxc6. lipid metabolism-associated cell membrane molecules (LMACMs) Pap2c, Lpcat3, Lrp8, Stab1 and ion transport proteins (e.g. Slco4a1, Slc30a5, Mcoln3) in addition to the putative efflux cell membrane receptor (PECMR) Mfsd10 is also unique to microglia compared to macrophages (32, 33). Transcriptional level studies of microglia in human and mouse brains by single-cell RNA sequencing identified P2RY12/13, SLC2A5, CX3CR1 and TMEM119 as the most abundant genes (34). In human brain, microglia express P2RY12/13, TMEM119, and CD11b, but express CD45 at a lower level, whereas exogenous macrophages express CD45 and CD11b,with TMEM119 expression remaining significantly characterized throughout the growth of microglia (35). In addition Buttgereit A et al. also identified Sall1 as a microglia signature gene (36). Therefore, we generally consider that the core features of microglia in the brain consist of CX3CR1, TMEM119, P2RY12, etc., and considered them as microglia cell-specific surface markers (35, 37, 38).

Sankowski R et al. found by combined high-throughput experiments that gene expression in tumor-associated microglia was out of homeostasis, with down-regulation of expression of core features and up-regulation of expression of pro-inflammatory and metabolic genes involving SPP1, APOE, CD163 and several type I interferon genes. This high expression was also corroborated by CyTOF (Cytometry by Time-Of-Flight) technique, including upregulation of CD163, TREM2, APOE, HLA-DR and GPR56 (53). In addition, microglia-specific inhibition of Itga4 (CD49D) allows it to also serve as a marker for differentiation between microglia and BMDMs in the context of glioma (39). CD163 expression tends to be positively correlated with patient OS, however the microglia marker CX3CR1 lacks such correlation. Therefore, we can consider CD49D and CD163 as BMDMs markers (40). Fluorescent labeling of them using red fluorescent protein (RFP) and GFP, i.e. macrophages (CCR2-RFP) and microglia (CX3CR1-GFP), can also distinguish to some extent between the two cell subpopulations of microglia and BMDMs (41). However, in the context of glioma, CX3CR1 may no longer be a specific marker of microglia, as Feng X et al. indicated that exogenous monocyte-derived macrophages can also express CX3CR1 (42). This was also confirmed by the study of Yona S et al., which found that blood monocytes express CX3CR1, and its expression elevates when they differentiate into macrophages (43). In HGG, tumor cells can lead to local inflammation that results in the disruption of the integrity of the BBB and leads to monocyte infiltration into gliomas and differentiation into TAMs that are indistinguishable from microglia in the tumor microenvironment, which is one of the possibilities that led to conflicting results in a series of previous studies (23).

Therefore, the current studies aimed at distinguishing microglia from BMDMs cell subpopulations have several limitations. For instance, most studies do not trace the origin of macrophages recruited to the tumor site any further and cannot demonstrate that their target macrophages are single-derived monocytes of exogenous origin. Furthermore, no study has proposed one or more specific markers with absolute convincing power to distinguish between the two. This suggests that in order to distinguish more clearly between the two cell subpopulations, we may need to iterate the current experimental model to find a more realistic experimental approach in humans.

### 2.4 M1/2 polarized phenotype

Researchers usually characterize macrophages by their bipolar differentiation status, with M1/2 phenotypes are deemed “pro-inflammatory” and “anti-inflammatory”, respectively. This dichotomous approach is widely used in macrophage typing (44). The M2 phenotype can also be subdivided into M2a, M2b and M2c polarization states (54). Microglia, although they have a macrophage-like polarized expression profile, may not be a true binary classification (45). This simplified dichotomy was also followed in several current studies based on TAMs, but transcriptomic analysis in microglia failed to demonstrate this relationship *in vivo* (46). Therefore, it is controversial whether microglia also exist in this polarized state *in vivo* (47). Genome-wide expression analysis of TAMs and comparison with expression data of M1, M2a, M2b and M2c polarized macrophages showed only a small overlap. Therefore, a simple dichotomy of M1/2 polarization may not be fully applicable for TAMs and can only be used as a reference classification method to facilitate the use of subsequent studies (48). Experiments by Müller S et al. proven that markers of M1 or M2 activation are able to be expressed simultaneously in the majority of microglia (49). And considering the complexity of transcriptional programming of microglia in gliomas, it goes beyond the simple division of M1/M2 polarization (30). Maas SLN et al. performed the isolation of cell subpopulations with palmitoylated fluorescent protein labeling and fluorescence-activated cell sorting (FACS) and analyzed their transcriptome. The results showed that as microglia approached the tumor, their anti-tumor genes were down-regulated while pro-tumor and immunosuppressive related genes were up-regulated. Therefore, they proposed a more precise scheme than M1/2 polarization classification, which classifies microglia by their proximity to the tumor (55).

## 3 Interactions between microglia and glioma

**Figure 1:**
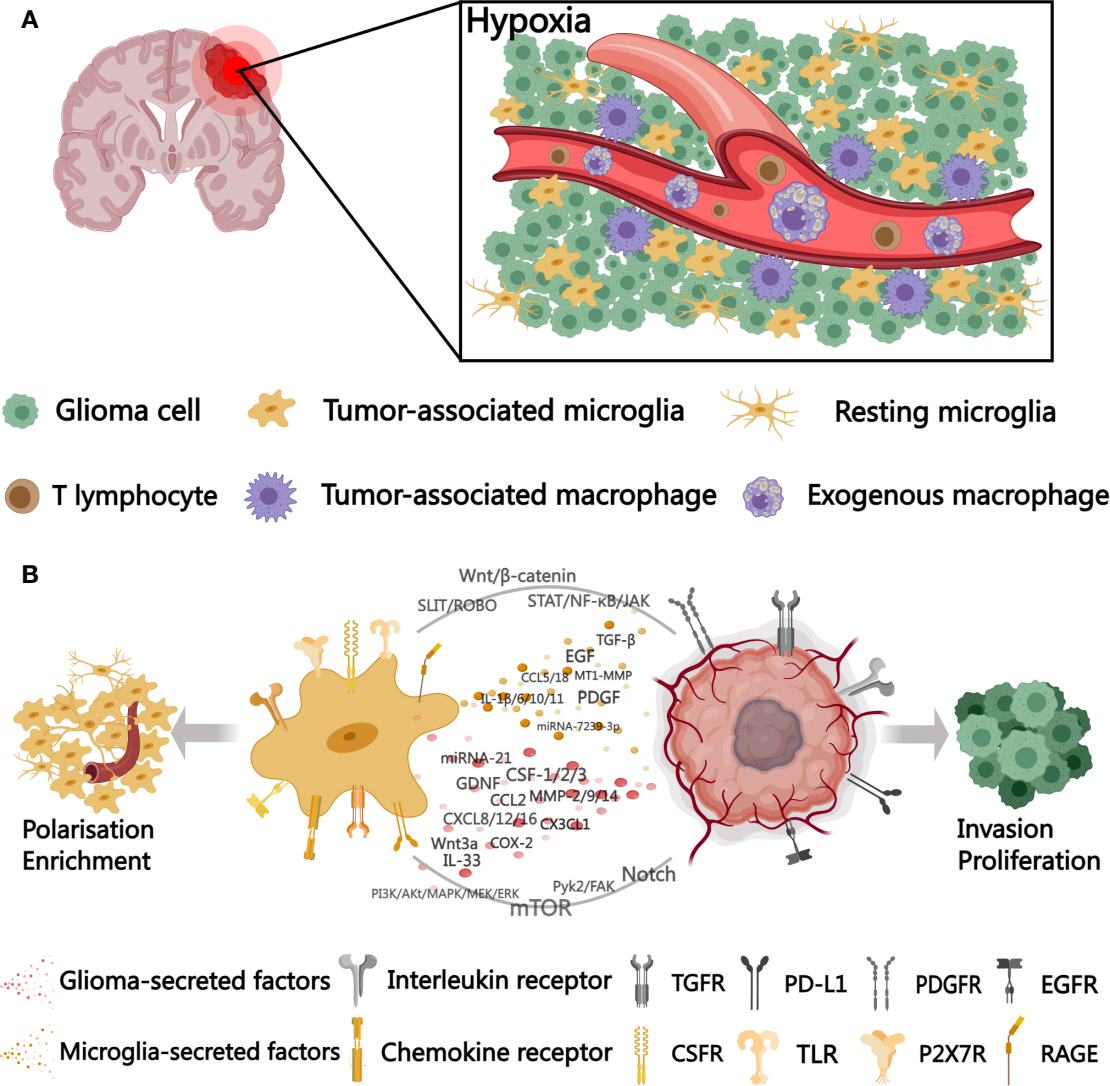
A schematic diagram outlining microglia-glioma cell interactions in the tumor microenvironment. **(A)** distribution of microglia and BMDMs in the hypoxic tumor microenvironment. **(B)** cytokines, signaling pathways, and surface receptors involved in microglia-glioma cell interactions. TGFR, transforming growth factor receptor; PD-L1, programmed cell death 1 ligand 1; PDGFR, platelet-derived growth factor receptor; EGFR, epidermal growth factor receptor; CSFR, colony-stimulating factor receptor; TLR, Toll-like receptors; P2X7R, P2X Purinoceptor 7; RAGE, receptor for advanced glycation endproducts. Created with MedPeer (www.medpeer.cn).

The coexistence of microglia promotes earlier glioma cell migration and enhances tumor growth by several-fold. A similar effect was observed in microglia conditioned medium, so Bettinger I et al. suggest that this phenomenon is induced by microglia-secreted substances and is unique, since glioma cell migration is only weakly stimulated in the context of oligodendrocytes and endothelial cells. In addition mediators secreted by tumor cells can also activate microglia leading to a further increase in motility (11). We broadly depict the interactions between microglia and gliomas in **Figure 1**.

### 3.1 Hypoxia

Under hypoxic conditions, glioma cells enhance glycolysis to deliver more lactate into the tumor microenvironment (56). Microglia exposure to lactate leads to a significant upregulation of MCT1 gene expression, where MCT1/2 are mainly accountable for lactate uptake (57). The recombinant protein of insulin-like growth factor binding protein 6 (IGFBP6), regulated by lactate levels in tumors, regulates microglia polarization toward the M2 phenotype, in addition to enhancing the capability of tumor cells to migrate and aggregate (58). However, in malignant tumors, enzymatic reactions, called “aerobic glycolysis” or “Warburg Effect”, continue to occur even under conditions of sufficient oxygen, as a result of hypoxia-induced transcription factors HIF-1/2, referred to as “pseudohypoxia” (59). In glioblastoma due to abnormal tumor angiogenesis leads to collapse of some of the proto-microvessels and formation of hypoxic Pseudopalisades regions. Microglia in hypoxic Pseudopalisades exhibit a specific pattern of movement along the fibers of glioma cells and show high phagocytic activity at the necrotic border of Pseudopalisades. The recruitment of microglia to the hypoxic niche is facilitated through HIF-1α downstream targets, and their motility is curtailed by hypoxia and shifted toward a pro-tumor phenotype. Hypoxic microglia infiltrating tumors promote tumor angiogenesis with HIF-1α-mediated upregulation of VEGF-A expression. In addition, macrophage migration inhibitory factor (MIF) is secreted to bind to CXCR4 on tumor cells to activate the Akt pathway to promote angiogenesis (60, 61). Glioma cells, stimulated by CD74, also secrete MIF and inhibit their IFN-γ secretion by phosphorylating the ERK1/2 pathway in microglia, inhibiting the shift of microglia to an anti-tumor phenotype (62). Interaction of tumor cells with microglia leads to high expression of LPA (Lysophosphatidic acid) and ATX (Autotaxin, an enzyme that synthesizes LPA) in microglia, supporting glioma progression and invasion, which is further enhanced in the hypoxic tumor microenvironment (63).

### 3.2 Epigenetic modification

Epigenetic mechanisms can convert cellular signals into enduring cellular responses through phosphorylation, acetylation and DNA methylation, the effects of these epigenetic modifications are mediated by modifications to histones and chromatin (64). Pretreatment of microglia with glioma-conditioned medium (GCM) strongly reduces lipopolysaccharide (LPS)-induced inflammatory gene expression (c-Myc, Mark1), whereby microglia acquire “transcriptional memory”. Hdacs-mediated histone modifications induce microglial polarization, in which HDACs 5 and 9 are the main players. Therefore, histone deacetylase inhibitor (HDACI) can eliminate tumor-induced “transcriptional memory”, block glioma-induced microglia polarization, and restore microglia responsiveness to LPS and inflammatory gene expression (65). In contrast, it was found in microglia conditioned medium (MCM) to promote apoptosis in glioma cells, exhibiting extra cytotoxic effects especially in response to LPS or IFN -γ stimulation. This killing may also be caused by epigenetic modifications mediated by increased secretion of histone proteinase B and nitric oxide (66). Glioma-induced transformation of microglia to a pro-tumor phenotype is also linked to increased acetylation of H4K16 in microglia and enhanced nuclear translocation of the deacetylase SIRT1 (67). pHGGs, including glioblastoma and DIPGs, and pediatric and juvenile HGGs are unique in that H3-K27M mutation occurs in more than half of cases, and the activity of EZH2 (histone-lysine N-methyltransferase Enhancer of zeste homolog 2) is suppressed by the H3-K27M mutation (68). EZH2 is a histone methyltransferase (HMTs) that regulates normal cellular physiological functions by catalyzing the methylation of H3K27 (histone H3 lysine 27) to control the expression of various genes. EZH2 is involved in glioblastoma-induced immunosuppression, it induces IL-10 as well as TGF-β secretion in glioblastoma cells and attenuates microglia phagocytosis and shifts toward the M2 phenotype (69). Lily Keane’s team proposed the notion that inhibition of EZH2 in tumor cells could not have a practical effect, whereas inhibition of EZH2 in microglia may have a beneficial effect. Because EZH2 mediates the inhibition of MHC I/II in microglia, inhibition of EZH2 in microglia restores some of the immunogenicity of the tumor and reinforces the sensitivity to ICB (immune checkpoint blockade). Tazemetostat, an epigenetic drug EZH2 inhibitor currently used for the treatment of lymphoma (Tazemetostat) may be tried as a therapeutic agent for glioma (70).

### 3.3 Signaling pathway

Self-associated molecular patterns (SAMPs) acts as a modifying factor in tumor cells, block immune responses in their tumor microenvironment and promote the development of immunosuppression. This immunosuppression is primarily mediated by PRRs (pattern recognition receptors) expressed in microglia that recognize DAMPs (damage-associated molecular patterns)and PAMPs (pathogen-associated molecular patterns) (71). DAMPs induces microglia in the area surrounding tumor necrosis to express IL-1β in a TLR-mediated manner, which induces glioma cells to release a range of cytokines such as CXCL8,CCL2, IL-1β and IL-6. Among them, STAT3 and NF-κB pathways are strongly activated so that IL-6 and CXCL8 act synergistically to promote the proliferation of tumor cells (72). Glioma cells secrete S1P (sphingosine-1-phosphate), which functions as an intercellular signaling molecule that recruits microglia to the tumor region and transforms them into a tumor-supportive phenotype by suppressing inflammation mediated by the NF-kB pathway (73). IL-11 secreted by microglia activates STAT3 signaling pathways, leading to increased MYC expression, and MYC overexpression transforms glioma cells into a stem cell state, thereby enhancing tumorigenicity and TMZ resistance (74). The interaction between microglia and tumor cells is a crucial factor that initiates the secretion of IL-6, which leads to hyperpermeability of brain endothelial cells through activation of the JAK (Janus Kinase)/STAT3 pathway, ultimately leading to enhanced BBB permeability, and BBB damage is a critical step in the progression of brain tumors (75). Moreover, PDIA3 (protein disulfide isomerase A3) is expressed on both glioma cells and microglia, where the expression level is higher in microglia than in microglia in tumor peripheral tissues. PDIA3 expression in tumor cells promotes microglia polarization toward the M2 phenotype and releases mediators such as CCL2 and COX-2 to activate the JAK/STAT pathway (76). Damage to the BBB causes aseptic inflammation, causing infiltration of microglia and transition to a pro-inflammatory polarized phenotype (77).

The mTOR (mammalian target of rapamycin) signaling pathway is activated in multiple human neoplasms, which is involved in the crosstalk between microglia and glioma. Activation of the mTOR pathway within glioma-associated microglia is about twice as high as in peripheral tissues. In an *in vitro* model of glioma at all stages, pharmacologic blocking of mTOR decreases urea and ARG1 levels (considered a biomarker of the M2 polarization state of microglia), decreases the M2 polarization phenotype of microglia by 40% and increases the proportion of the M1 phenotype realizing its direct antitumor effects (78). It has been shown that glioma progenitor cells enhance mTOR signaling in microglia but not in BMDMs via the PI3K (phosphatidylinositol 3 kinase)/Akt (Protein Kinase B) axis. mTOR-mediated control of STAT3 and NF-κB activities promotes an Immune-suppressive microglial phenotype that impedes the proliferation, infiltration, and immunological responses of effector T cells, thus facilitating tumor immune escape and growth (79). Microglia releasing EGF binds to EGFR in glioblastoma activating its downstream PI3K/Akt/mTOR signaling pathway (80, 81). ZDHHC-type palmitoyl acyltransferases, which are aberrantly expressed in gliomas, promote glioma advancement and microglial invasion through the PI3K/Akt pathway. Blockade of ZDHHCs with 2-BP (2-bromopalmitate) promotes apoptosis of tumor cells and also promotes TMZ sensitivity of glioma cells (82). SLIT2 induces migration of ROBO1/2 (Roundabout1/2) receptor-expressing cells, and in malignant gliomas, SLIT2 expression increases with malignancy. SLIT2 promotes microglia chemotaxis and polarization toward a pro-tumor phenotype through ROBO1 and ROBO2-mediated activation of the PI3K pathway. SLIT in the SLIT-ROBO signaling axis can regulate cell motility mediating behaviors associated with tumor aggressiveness by regulating actin and the microtubule cytoskeleton.Tumor growth can be inhibited by inhibiting SLIT2 signaling (83, 84).

It is well known that the Wnt/β-catenin signaling pathway is implicated in the regulation of cell proliferation, migration and apoptosis, and promotes cancer progression (85). CCN4, also referred to as WISP1 (Wnt1-inducible signaling pathway protein-1), is secreted by GSCs to bind with integrin α6β1, thereby activating the Akt pathway to enhance GSC proliferation and microglia viability, creating a tumor-supportive cellular environment (86). *In vitro* experiments demonstrate that glioma cell-derived Wnt3a triggers the activation of the Wnt/β-catenin pathway in microglia, induces them to express ARG-1 and STI1, shifts them to the M2 phenotype, enhances tumor infiltration, invasion, growth and the crosstalk between microglia and gliomas (87). The proto-oncogene AEG-1, overexpressed in gliomas, is targeted to GSK-3β (glycogen synthase kinase-3β) to activate the Wnt/β-catenin pathway, mediating a pro-tumor phenotypic shift in microglia and reducing the sensitivity of glioma cells to TMZ (88). This also demonstrates that the Wnt/β-catenin pathway is also markedly upregulated in glioma cells. In addition to eliciting a phenotypic shift of immune-suppressive microglia to anti-tumor growth, inhibiting Wnt signaling also exerts a direct inhibitory effect on the growth of glioma cells (89).

CECR1,Cat Eye Syndrome Critical Region Protein 1, modulates microglia polarization and M2-type microglia exhibit sustained high expression of it, especially in malignant glioma. CECR1-induced paracrine effects in M2-type microglia activate MAPK (mitogen-activated protein kinase) signaling and stimulate migration and proliferation of glioma cells (90). Glioma cells activate the MEK (A kinase of MAPK)/ERK (extracellular regulated protein kinases) signaling pathway by secreting cytokines that downregulate the expression of aquaporin 1 (AQP1, a transmembrane glycoprotein involved in tumor angiogenesis) on microglia, attenuate their response to pro-inflammatory factors, and convert them to a pro-tumor phenotype, ultimately promoting tumor progression. The MEK inhibitor Trametinib, an FDA-approved drug for the treatment of lung cancer, inhibits this downregulation of expression, blocks the conversion of microglia to a pro-tumor phenotype, and inhibits glioma growth (91). Tumor-associated microglia promoting glioma cell invasion can be mediated by CSF-1R signaling on gliomas, a pathway involving CSF-1 signaling via ERK that upregulates the expression of dual-regulatory proteins (AREG) in microglia, which are ligands for epithelial growth factor receptor (EGFR) (92). The release of various cytokines such as EGF, PDGFβ and SDF-1α (CXCL12) from microglia activates the Pyk2 and FAK signaling pathways, thereby promoting proliferation and invasion of glioma cells (93). Glioma cells can maintain their own sustained proliferation by reducing Notch signaling, significantly inhibiting the recruitment of anti-tumor immune cell populations, contributing to the formation of anti-inflammatory microglia, and contributing to the immune escape microenvironment of glioma. Furthermore, as a result, gliomas will also gain resistance to microglia re-educating therapy (94).

### 3.4 Surface function receptors

#### 3.4.1 Chemokine and chemokine receptor

Chemokines can directly stimulate the migration of diverse immune cells such as microglia in the TME, and May also enhance tumor progression and migration by directly affecting tumor cell proliferation and indirectly by regulating neovascularization and recruiting immunosuppressive cells. The crosstalk between microglia and glioma mainly involves the CC, CXC, and CX3C chemokine families. Chemokine receptors represent a heterogeneous group of GPCRs (G protein-coupled receptors) that tend to be expressed systemically, most commonly on immune cell populations and driven by inflammation. In addition, non-immune cells like endothelial cells are also capable of expressing it (95).

##### 3.4.1.1 CC family

The gene expression of CCR1 receptor and its ligand was strongly upregulated in microglia exposed to glioma conditioned medium probably mediated by CSF-1R signaling, including CCL3/5/6/9,especially CCL3.However, the current study proposes that the CCR1 signaling axis may not be a major factor in microglia chemotaxis (96). Previously, it was generally believed that only macrophages expressed CCR2, but now studies have found that microglia in gliomas can also express it. In a mouse model of CCR2-knockout microglia, it was found that CCR2 deficiency reduced pro-inflammatory molecule expression, phagocytic activity, and migratory capacity and affected the degree of infiltration (97). However, a study by Mizutani M et al. suggested that CCR2 may not be expressed in the mouse model (98). CCL2, referred to as MCP-1 (Monocyte chemotactic protein-1), is secreted by glioma cells and binds to CCR2 expressed on microglia, inducing microglia to recruit to the glioma region and release interleukin-6 (IL-6) thereby promoting the invasion and growth of glioma cells (99, 100). CCL20 secreted by glioma cells induces CCL2 secretion by peritumor microglia (PGAM) and attracts a variety of peripheral immunosuppressive cells via CCR2/4 to participate in the establishment of the immunosuppressive microenvironment of glioma, which includes MDSC (myeloid-derived suppressor cells) and Tregs (regulatory T cells) differentiated from monocytes (101). This allows us to antagonize CCR2 for antitumor purposes, and studies have also confirmed that CCX872, a CCR2 antagonist, increases MS in animal models and further improves MS and OS when used in combination with anti-PD-1 (102). GM-CSF -induced CCL5 secretion by microglia may increase the level of glioma infiltration when tumors progress. The CCL5-CCR5 pathway has been demonstrated to promote tumor invasiveness by activating intracellular calcium cascade (increased calcium levels) and CaMKII (Calcium/Calmodulin Dependent Protein Kinase Kinase II)-dependent matrix metalloproteinase 2 (MMP2) upregulation in tumor cells (103). Venkataramani et al. observed that elevated calcium levels in glioblastoma cells also lead to the re-formation of glioblastoma microtubules thereby causing increased tumor aggressiveness (104). CaMKII has been found to be a contributing factor to ICB resistance by promoting CD8^+^ T cell depletion and reducing the proliferation of effector CD4^+^ T cells. And TAMs show high expression levels of CaMKII, which is often linked to a worse prognosis in patients with glioblastoma (105). Blocking the CCR5 receptor reduces microglia migratory capacity and blocks microglia M2 polarization and shift to M1 phenotype by inhibiting AKT pathway and mTOR pathway, respectively. So MRV (Maraviroc), a CCR5 antagonist, has potential as a pharmacological inhibitor (106). In Addition, microglia polarized to the M2 phenotype and upregulated their CCL18 expression in response to IL-4 stimulation. In contrast, CCL18, a chemokine expressed only in the cellular context of humans, is often linked to poor patient prognosis when its high expression inhibits the recruitment and maturation of cytotoxic cells, like dendritic cells or lymphocytes, and disrupts their immune competence, and shows capacity for binding to the CCR8 on glioma cells and promote tumor progression via ACP5 signaling (107, 108).

##### 3.4.1.2 CXC family

PDCD10 (Programmed cell death 10) overexpression in glioblastoma promotes microglia recruitment to the tumor core through elevating cell migration capacity and evokes microglia polarization toward a pro-tumor phenotype with CXCL2-CXCR2 axis signaling (109). Microglia and glioma cells secrete stromal-derived factor-1 (SDF-1 or CXCL12), a microglia recruitment molecule that normally binds to its receptor CXCR4, a specific receptor for CXCL12 expressed on microglia, to form the CXCR4-CXCL12 axis that attracts microglia to hypoxic regions of the tumor and mediates tumor proliferation, abnormal angiogenesis, invasion and drug resistance (110–112). CXCL14 secreted by PXA (pleomorphic yellow astrocytoma) cells binds to CXCR4, participates in the construction of the TME, and mediates the tropism of CD8^+^ T cells in a proportionate manner to the dose, in which the existence of a large number of activated microglia is also found (113). Glioma-secreted CXCL16 binds to CXCR6 on microglia, and the CXCL16-CXCR6 axis mediates the polarization of microglia, promoting a shift to a pro-tumor phenotype and promoting tumor invasion and progression (114).

##### 3.4.1.3 CX3C family

CX3CR1 expression is mainly restricted to microglia, whereas CX3CL1 expression is mostly confined to neurons, and co-expression of CX3CR1 and CX3CL1 promotes microglia aggregation and increased tumor microvessel density through the mediation of CCL2 and matrix metallopeptidase 9 (MMP9) upregulation, contributing to tumor progression and shifting LGG’s to a higher malignant grade (115). In Nf1 optic gliomas, TAMs are mostly CD11b^high^ CD45^low^, and in mouse models with downregulated CX3CR1 expression, yet exhibit delayed tumor growth (116). In contrast, CX3CR1 deficiency causes a dose-dependent overexpression of IL-1β by microglia. This activates the p38 MAPK pathway, upregulation of CCL2 expression, and enhancement of the tumor stem cell (GSC) phenotype, promoting glioma progression (42).

##### 3.4.1.4 Olfactomedin-Iike 3

In glioblastoma, CLOCK and its heterodimeric chaperone BMAL1 behave as tumor-promoting factors, directly modulating the NF-κB pathway, thereby regulating glioma cell migration and proliferation, and maintaining GSC stemness. In addition, it enhances the transcriptional expression of OLFML3, a recently discovered chemokine that recruits immunosuppressive microglia to the TME to trigger pro-tumor immunity (117). Olfml3 is also a immediate target gene for all TGF-β isoforms in mouse microglia and is a crucial factor in TGF-β-induced pro-tumor phenotypic transformation of microglia, and also promotes tumor progression by increasing tumor cell invasion and migration capacity. In TME microglia-derived OLFML3 a pro-angiogenic factor distinct from VEGF (vascular endothelial growth factor), which promotes brain microvascular endothelial cell migration and germination mainly through activation of the SMAD1/5/8 signaling pathway (117, 118).

#### 3.4.2 Receptors associated with tumor angiogenesis

##### 3.4.2.1 Platelet-derived growth factor receptor

Significant upregulation of PDGF receptor (PDGFR) expression is a recognized marker of aberrations in the glioblastoma subgroup. Glioma cells express PDGFRA, while PDGFRB is predominantly localized in the glioma-associated extracellular matrix. M2-polarized microglia, rather than BMDMs, provoke PDGFRB expression in glioma cells and stimulate their migratory ability, and have a tight interaction with pericytes for intra-tumor angiogenesis. It recruits pericytes into the tumor microvascular system, not in combination with perfused vessels, but in the PDGF signaling pathway that mediates tumor progression (119).

##### 3.4.2.2 Neuropilin-1

The transmembrane receptor Nrp1 is a co-receptor for VEGF expressed on microglia that, when activated, drives microglia to migrate toward tumor areas and polarize toward the M2 phenotype, and correlates with tumor neovascularization and immunosuppressive microenvironment formation (120). In general, early microglia dominate the tumor, but with increased neovascularization, BMDMs becomes the main infiltrating cell population. However, microglia with downregulated Nrp1 can lead to reduced tumor neovascularization, preventing BMDMs from infiltrating the tumor and eventually reasserting a dominant position (121).

##### 3.4.2.3 Macrophage scavenger receptor 1

CD204-positive microglia typically accumulate around tumor vessels and in necrotic areas and are in close proximity to podoplanin+ (PDPN) stem cell-like glioblastoma cells. The number of CD204-positive microglia increases with glioma grade and tends to exhibit a pro-tumor phenotype. Furthermore, in gliomas with high CD204 expression, upregulation of genes related to angiogenesis often forecasts a negative prognosis for patients (122, 123).

##### 3.4.2.4 Receptor for advanced glycation end products

The binding of RAGE receptors in glioma-associated microglia to various ligands like advanced glycation endproducts (AGEs), S100 proteins and high mobility group box 1 (HMGB1) mediates tumor angiogenesis and promotes glioma invasion and progression (90).

##### 3.4.2.5 Cannabinoid receptor

Cannabinoid binding to the CB1R/CB2R inhibits the growth of gliomas. Among them, CB2R expression directly correlates with the malignancy of gliomas, and the selective CB2 agonist JWH133 induces glioma cell apoptosis to induce tumor regression (124). However, one study suggested that JHW133-treated microglia would shift to an M2 immunophenotype with detectable overexpression of CYP2J2, which metabolizes more arachidonic acid in microglia to 11,12-EET (epoxyeicosatrienoic acid), of which 11,12-EET significantly promotes human brain microvascular endothelial cells (HBMECs)-mediated tumor angiogenesis, increasing cancer cell proliferation and preventing apoptosis, thereby promoting glioma progression (125, 126).

#### 3.4.3 Purinergic receptor

P2X7R was highly expressed on glioma-associated microglia and higher than on peritumor microglia, and the quantity of P2X7R was directly linked to the malignancy of glioma. P2X7R expression is also observed on glioma cells and may mediate the regulation of the immune phenotype of microglia and play a tumor-supporting and nutritional role. However, until now, there has been little progress in the development of P2X7R-targeted drugs in glioma. The application of P2X7R agonists and antagonists, such as OxATP, BBG and BzATP, has yielded varying outcomes in glioma models of both animal and human origin. AZ10606120, a potent antagonist of P2X7R, significantly inhibited tumor growth directly through microglia inactivation. It also demonstrated better effects than TMZ, the P2X7R agonist BzATP, and its inhibitors BBG and OxATP (127, 128). In zebrafish models studying brain tumors, high calcium levels in cells can be monitored and dynamically regulated, and calcium-stimulated ATP and P2RY12 signaling mediates the interaction of tumor cells with microglia, ultimately promoting their own proliferation (129). And it has been proposed that the swift closure of the BBB is dependent on the P2RY12-driven chemotactic response of microglia after BBB damage (130). Gliomas, in contrast to other malignancies, are more dependent on cell motility than blood circulation for their invasion and spread, and are therefore more regulated by calcium-dependent signaling in tumor progression. Apparently, not only calcium signaling is regulated in tumor cells, but calcium signaling in microglia is responsible for their activation and motility, especially their P2Y2 receptors, which have an important role in microglial cell chemotaxis (131). The morphological transition and motility patterns of microglia are regulated by purinergic signaling and mediated mainly through P2Y1, P2Y12 and P2Y13 receptors (132). In addition, some P2Y receptors are also implicated in the interplay between microglia and tumors, as an illustration, if selective stimulation of P2Y14 receptors on microglia reduces IL-6 secretion and even tumor cell proliferation (133).

#### 3.4.4 Toll-like receptors and matrix metalloproteinase

Glioma cells secrete MMP-2 in an inactive state, which requires cleavage to attain an active form, microglia express the enzyme that cleaves MMP-2, MT1-MMP. In the normal brain, microglia generally do not express this enzyme or show only very low expression in cerebral white matter. However, it can be induced by endogenous ligand versican of TLR2 secreted by tumor cells in close contact with tumor cells, and its own TLR2 is activated to induce MT1-MMP expression, accompanied by up-regulated expression of MMP-9, and the magnitude of expression is directly proportional to the malignancy of glioma (134–136). In addition to versican ligands, TLR2-expressing microglia are also activated by glioma-released mediators, such as HMGB1 (high mobility group protein B1), HSP (heat shock protein), and hyaluronic acid stimulation leading to MT1-MMP upregulation, enhancing ECM restructuring which in turn enhances TLR2 signaling, ultimately promoting glioma expansion and invasion, constituting a vicious circle of microglia-glioma crosstalk (137). Minocycline inhibits glioma invasion and progression by attenuating MT1-MMP expression in microglia (138).

However, while minocycline was employed as an adjunctive therapy in clinical trials (NCT02770378, NCT02272270 and NCT01580969), it did not demonstrate actual clinical effects (139). Glioma cells release GDNF (Glial cell line-Derived Neurotraphic Factor), a chemotactic agent that recruits microglia to the tumor region and also induces a shift to a pro-tumor phenotype. This is accompanied by an upregulation of its TLR1 and TLR2 expression and mediates an increase in MMP-9/MMP-14 expression, promoting microglia-mediated glioma progression (140, 141). MHC class II expression is suppressed by TLR2 activation on microglia, limits CD4^+^ T cell-dependent antitumor immunity, diminishes the antigen presentation ability of microglia, and enhances the immunosuppressive profile of the tumor microenvironment (142). After microglia TLR3 and TLR9 are co-activated, microglia shift to an anti-tumor phenotype and enhance migratory activity to accumulate in glioma tissue, which in turn inhibits glioma cell function by releasing cytokines or directly kills glioma cells by enhancing phagocytic activity of microglia (143). Microglia tend to be highly plastic and are usually transformed to an anti-tumor M1 phenotype mediated by TLR4 activation and IFN-γ stimulation (144). The let-7 miRNA family with conserved sequences is an immunomodulatory factor expressed widely in the brain and is involved in glioma development and progression (145). Let-7 miRNA directly activates TLR7 in microglia, leading to upregulation of MHC I and ICAM1 (CD54) and induction of TNF-α production to inhibit glioma growth (146). Activation of TLR5 in microglia and mediates the release of multiple inflammatory molecules causing inflammatory neuronal damage in the CNS, but in contrast to TLR 2/3/4/7/9, TLR5 is not involved in microglia-glioma interactions (147).

### 3.5 Cytokine

#### 3.5.1 Colony-stimulating factor

CSF-1 secreted by glioma cells acts on CSF-1R on microglia, which attracts microglia to the tumor and induces a shift to a pro-tumorigenic phenotype, enhancing the crosstalk between microglia and glioma. Simultaneously, stimulating microglia to express EGFR also facilitates tumor cell invasion and growth (80). Nijaguna et al. demonstrated that glioma-secreted CSF-1 stimulates microglia to upregulate IGFBP1 expression levels and that upregulation of IGFBP1 underlies tumor angiogenesis (148). Colony-stimulating factor 2 (CSF-2), which is also called GM-CSF, is a glioma-derived CSF-2 that attracts and induces microglia to polarize into a pro-tumor phenotype. When it is knocked down or depleted, a decrease in glioma cells and apoptotic microglia are found (149). In contrast, in a rat model, stimulation with G-CSF (CSF-3) enhanced microglia proliferation and infiltration of tumors, accompanied by a decrease in TGF-β and IL-10 production and enhanced antigen expression, ultimately improving survival in rats transplanted with hypodifferentiated malignant gliomas by altering the tumor microenvironment (150).

#### 3.5.2 Interleuki family

Human interleukin-33 (IL-33), belonging to the IL-1 family, is a cytokine that mediates endogenous cellular alerts and exerts a tumor-promoting effect in various types of tumors, gliomas included (151). IL-33 exists in nuclear and secretory forms; nuclear IL-33 stimulates glioma cells to express inflammatory cytokines (IL-1β, IL-8, IL-6, etc.), and secretory IL-33 is secreted by glioma cells, which activates microglia and drives them to a pro-tumor phenotype; both synergistically accelerate glioma progression (152).

IL-10 secreted by microglia in gliomas is an immunosuppressive cytokine and correlates with the malignancy of gliomas (153). Concurrently, glioma cells can also secrete the immunosuppressive factor interleukin-10 (IL-10) via an autocrine or paracrine pathway and microglial PD-L1 expression is elevated, thus promoting the formation of an immunosuppressive tumor microenvironment (154). In addition, GSC also induces IL-10 secretion from microglia and polarizes microglia to the M2 phenotype, inhibiting microglia phagocytosis (155).

#### 3.5.3 Transforming growth factor-β

TGF-β is an immunosuppressive factor in gliomas and patient prognosis is linked to the level of its expression. Tumor tissues expressed both TGF-β1 and TGF-β2 isoforms of TGF-β. TGF-β1 secreted by glioma cells activates microglia in tumors and secretes IL-1β through lipoprotein E (ApoE)-mediated NLRP1 inflammasome. TGF-β2 affects brain tumor-initiating cells (BTIC) proliferation and migration and stimulates tumor angiogenesis thus exerting an immunosuppressive function, and the two TGF-β isoforms synergistically promote tumor progression (156, 157). TGF-β1released from tumor-associated microglia promotes the expression of MMP-9 in GSC and maintains the characteristics of glioma stem cells, enhancing the aggressiveness of gliomas (158). Targeted blocking of TGF-β presents a promising therapeutic approach, but it is currently found to be less feasible because of its severe side effects, resulting in acute inflammation and disturbance of immune system balance (159). Liu H et al. proposed that depletion of TGF-β1/TGFBR1 (TGF-β type I Receptor) could reduce the concentration of microglia within tumors and suppress tumor growth (157). The better prognosis of patients with simultaneous low expression of TGFBR1 and PD-L1 is in some agreement with the above-mentioned studies, making the combination of TGF-β1/TGFBR1 inhibitors and PD-1/PD-L1 inhibitors a potential immunotherapeutic strategy for glioma (160). Furthermore, research conducted by Ye XZ and colleagues has indicated that knockdown of TGFBR2 (Transforming Growth Factor Beta Receptor 2) diminishes the invasive potential of glioma cells (158). The use of plasmid-transcribed small hairpin RNA (shRNA) to downregulate TGFBR2 expression also can achieve exactly this effect, i.e., to eliminate the glioblastoma invasive and migratory response induced by microglia-secreted TGF-β (159).

### 3.6 Functional protein

An often-observed mechanism of immune escape in tumors is the upregulation of CD47 (Integrin-associated protein, IAP) expression and evasion of immune surveillance by binding to SIRPα on phagocytes and inhibiting their phagocytic capacity (161). Elevated LRIG2 (Leucine Rich Repeats And Immunoglobulin Like Domains 2) gene expression in glioblastoma cells triggers CD47 upregulation and activates the CD47-SIRPα anti-phagocytic axis (162). CD47-SIRPα signaling prevents excessive microglia phagocytosis, ensuring that microglia engulf specific synapses and avoid others, maintaining normal developmental growth of synapses (163). Microglia were shown to act as effector cells against the action of the CD47-SIRPα axis even in the absence of a background of peripheral macrophages, and the phagocytic capacity of microglia was also stronger than that of macrophages in tumors. This demonstrates that microglia have an anti-tumor effect independent of macrophages (41).

Elevated expression of GPNMB (glycoprotein non-metastatic B) and SPP1 in microglia is linked to an unfavorable prognosis in glioblastoma patients (48). SPP1 (secretory phosphoprotein 1) also identified as OPN (osteopontin). In glioma perivascular ecotone, OPN promotes increased stem cell-like properties (HIF-2α-mediated) and radioresistance of neighboring tumor cells through activation of CD44 signaling and also have a synergistic effect on PDGF-induced aggressive growth of glioblastoma (164). CD44 is a transmembrane glycoprotein involved in intercellular or interactions with the matrix. CD44 in microglia is pivotal for the initiation of the TLR2 pathway and the upregulation of TNF-α, MMP9 and IL-1β expression (165). Additionally, integrin αvβ5 (ITGαvβ5) is highly expressed as a key receptor for OPN on tumor-infiltrating microglia, and the binding of both mediates the recruitment of glioma cells to microglia and polarization to the M2 phenotype, and reduces the direct cytotoxic sensitivity of tumors to CD8^+^ T cells (166). In gliomas, GSCs are also predominantly clustered in the perivascular niche and are usually surrounded by tumor-associated microglia, positively correlated with the cell density of microglia, suggesting that GSCs may be more adept at recruiting microglia than glioma cells (167).

Fasl is a type II transmembrane glycoprotein known as a death factor, and Fas-FasL binding mediates programmed cell death. Microglia represent the major origin of FasL expression in gliomas and are responsible for immune escape in the glioma microenvironment by promoting apoptosis of T cells that express FasL (168). Dcf1 (Dendritic cell-derived factor 1) also is a membrane protein that significantly contributes to the differentiation of neural stem cells. Available studies indicate that its overexpression in glioma cells can lead to reduced proliferation and invasive capacity of glioma cells, even mediate apoptosis of tumor cells by disrupting mitochondria (169). Dcf1 deficiency has a large impact on microglia function, promoting microglia activation but reducing their migration and phagocytosis (170). During tumor initiation, pre-neoplastic cells also can reduce microglia migration and phagocytosis by altering their Wasla gene and WASL expression, leading to a morphological shift of microglia to amoeboid morphology. WASL plays a critical role in actin cytoskeleton organization and is essential for maintaining microglia core activity during the initiation phase of glioma (171).

The interaction of glioma with microglia induces high expression of SELP and induces microglial polarization towards a pro-tumor phenotype through the SELP-PSGL-1 (P-selectin with P-selectin glycoprotein ligand-1) axis, enhancing their immunosuppressive capacity and promoting tumor progression. Recombinant SELP decreased the phagocytic activity of microglia and reduced the expression of nitric oxide synthase (iNOS) and the release of nitric oxide (NO). Anti-SELP therapy holds potential for enhancing tumor sensitivity to immunotherapeutic approaches and conventional radiotherapy treatments (172). Additionally, Stress-Inducible Phosphoprotein 1 (STIP1), abundantly present in glioma cells, and Stress-Inducible Protein 1 (STI1), released by microglia, significantly contribute to the progression of glioblastoma multiforme, with their expression levels positively associated with the degree of tumor malignancy (173).

### 3.7 Extracellular vesicles

The International Society for Extracellular Vesicles (ISEV) designates EVs as irreproducible microparticles composed of lipid bilayers that are naturally secreted by cells. The diameter of small EVs (sEVs) typically ranges below 200 nm and include the well known exosome and medium or large EVs (mEVs or lEVs) with diameters between 200 and 1000 nm (174). In addition to differences in size, the two subtypes differ in terms of their formation mechanisms and cargo. EVs in general can transport biologically active molecules like nucleic acids, lipids and proteins between cells, consisting of DNA and assorted RNA types (microRNA and mRNA) (175).

glioblastoma patients generally exhibit elevated levels of EVs in their plasma compared to healthy individuals, suggesting that EVs could play a role in the pathogenesis and advancement of gliomas (176). EVs, as substances that mediate intercellular communication, can execute diverse functions during glioma progression including promoting immunosuppression and immune escape, and tumor cells secrete about 10-fold more EVs than normal cells. The interaction between EVs and microglia is further intensified by the hypoxic and acidic milieu of the TME, inducing a shift toward an anti- or pro-tumor immune phenotype in microglia (177). This was confirmed in an *in vitro* model. Exposure of microglial cells to glioblastoma cell-derived EVs revealed an upregulation of the expression of the pro-tumor factors CXCL1/10, CCL2/CCL5 and IL-6 as well as an increase in their own proliferation. And Spellicy SE et al. also suggested that glioma-derived EVs may exert a selective influence on microglia, which is not evident in other immune cells such as BMDMs (178). Furthermore, overexpression of Cavin1 (polymerase Iand transcript release factor or PTRF) enhances the secretion of pro-tumor type EVs by glioma cells and demonstrates a direct association with the malignancy of glioma patients. Mediators such as IFN-γ/LPS, Wnt3a, and 5-hydroxytryptamine in the TME is able to trigger the release of EVs from microglia (174, 179). GSC transfer lncRNA MALAT1 (nuclear-enriched abundant transcript 2, NEAT2) cells into microglia in an EVs manner and regulate inflammatory responses mediated by the miR-129-5p and HMGB1 cascade to achieve pro-tumor effects (180). Secretion of EVs-mediated miRNA-21 transfer by glioma cells triggers the downregulation of particular mRNA targets in microglia, reprogramming microglia, with more prominent alterations in Btg2, thereby reducing cell cycle protein D1 activity to negatively regulate cell proliferation, leading to enhanced proliferation of microglia and tumor cells and promoting glioma progression (181). M2 phenotype microglia can promote glioma proliferation and migration by delivering miRNA-7239-3p to glioma cells via exosomes, regulating their tumor-associated protein expression (BMAL1 down-regulation, CLOCK up-regulation) and reducing tumor cell apoptosis (182). In contrast to this situation, microglia released sEVs after stimulation with LPS/INF-γ and IL-4 exhibit anti-tumor effects, in addition unstimulated microglia may release more sEVs and also reduce the invasion of glioma cells. This may be mediated by miR-124 in sEVs, causing a decrease in lactate, nitric oxide and glutamate in the microenvironment, inhibiting tumor neovascularization and tumor cell proliferation (183). Externally, miRNA-504 is overexpressed in GSCs and delivered to microglia via EVs, promoting their polarization to the M1 phenotype and exerting a bystander effect on surrounding microglia. In addition, miRNA-504 also inhibits angiogenesis and GSC stemness, which exerts a synergistic tumor suppressive effect (184). Under hypoxic conditions, glioma-derived exosomes mediated their pro-tumor effects by activating STAT3 and inhibiting NF-κB signaling, with miRNA-1246 being the most enriched and also highly concentrated in the CSF of patients (185). WT1 expression is notably elevated in EVs isolated from the CSF of patients with malignant glioma, and glioma-derived EVs with WT1 promote tumor progression by suppressing the expression of the Thbs1 gene(Thbs1 is a negative regulator) and TSP-1 (TSP-1 has anti-tumor angiogenic properties) production in microglia (186). From this, it is natural to think that monitoring EVs in blood or CSF could be a new attempt for clinical biopsy of glioma. One study attempted to detect CSF-derived EVs to diagnose EGFRvIII-positive glioblastoma with a sensitivity of over 60% and a specificity of over 95% (176). Given that EVs are pivotal in the interaction between glioma and microglia, it is possible to consider ways to inhibit tumor progression by modifying or selecting specific cargoes. For example, Fareh M et al. posited that the replication and tumorigenicity of glioma cells could be reduced by modifying them to sustain the expression of miR-302-367 and to be internalized by surrounding glioma cells through paracrine secretion (187). In addition, EVs-based drugs are thought to be effective through the BBB, and then EVs can be used as a platform to inject miRNA-124 into the TME to inhibit glioma cell proliferation and migration. Moreover, by targeting the STAT3 pathway, it can impede microglia polarization toward the M2 phenotype (188, 189).

## 4 Treatment

Patients with glioma often receive conventional radiation therapy after surgery, and although the dose of radiation therapy is positively correlated with patient prognosis, serious complications such as neuroinflammation, gliosis, cerebral atrophy, and cerebral hemorrhage can occur with increasing dose, where neuroinflammation and reactive gliosis are mediated by PGE2 secreted by microglia. In parallel, rising BBB permeability microglia are recruited to enhance the pro-inflammatory microenvironment (190). Despite numerous attempts over an extended period, the approval of new drugs for glioma treatment remains limited. The unique physiological structure of the BBB presents significant challenges to drug development (191). Therefore, until now, the conventional treatment of glioma is still limited to surgical resection, TMZ and radiotherapy, and there are very few other efficient treatment options available, so that the survival outcome for patients with glioma has shown little improvement in recent years. Hence, there is an urgent demand for the development of novel therapeutic approaches that can make a difference to the current situation, and targeted immunotherapy has become a popular area of research (192).We summarize potential antitumor therapies targeting microglia in **Table 2**.

**Table 2:** Potential treatments.

Program name	Type	Action target	Potential mechanism of action	Reference
**EV-based miRNA-124**	Engineering drugs	Microglia	Impede microglia polarization toward the M2 phenotype in the TME.	(188)
**PLX5622**	CSF-1R inhibitor	Microglia expressing PD-L1	Reduction of PD-L1 levels in microglia in combination with p38 MAPK inhibitor and PD-L1 antibody.	(193, 194)
**BLZ945**	CSF-1R inhibitor	Microglia (pro-tumor phenotype)	Reduced survival of M2 phenotypic microglia impairs their pro-tumor ability and ultimately inhibits tumor growth.	(195)
**PVSRIPO combined with PD-1/PD-L1 blockade**	Immunotherapeutic agents and OV	Microglia expressing PD-L1 and glioma cells	Removal of PD-L1-expressing microglia to enhance the antitumor effect of PVSRIPO.	(196)
**CL or CTX**	Macrophage scavenger or Cytotoxin drugs	Microglia	Clearance of microglia, which reduces the secretion of antiviral factors and ultimately improves the antitumor efficacy of OV.	(197, 198)
**Apigenin**	Flavonoids	Microglia	Reversal of M2-phenotyped microglia to M1-phenotype and enrichment toward the tumor region. (Dose-dependent and non-toxic.)	(199)
**Quercetin**	Flavonoids	Microglia	It modulates the chemotaxis of glioma cells to microglia, induces microglia proliferation and restores their original immune response to inflammation.	(200)
**PSPD3R (Rutin)**	Flavonoids	Microglia and glioma cells	In addition to these functions, it induces autophagy and apoptosis in glioma cells, thereby inhibiting tumor progression.	(201)
**ADI-PEG20**	Arginine-Depleting Agents	Microglia and glioma cells	Targeting arginine depletion and enhancing tumor sensitivity to radiotherapy. And it induces microglia to further infiltrate the tumor with an M1 phenotype for anti-tumor effects.	(202, 203)
**Therapeutic miR-155-like virus**	Nanotechnology	Microglia (pro-tumor phenotype)	It can reprogram microglia from M2 phenotype to M1 phenotype to achieve tumor suppression.	(204)
**siRNA CD73-loaded cationic nanoemulsion or rAAV2-IL-15 modified microglia**	Engineering Microglia and Nanotechnology	Microglia	Improving the immunosuppressed tumor microenvironment by blocking microglia recruitment and infiltration.	(205, 206)
**CAR-MΦs**	Engineering Microglia and Nanotechnology	Microglia	Generating CAR-MΦs in the surgical cavity to remove residual GSCs and prevent postoperative glioma recurrence.	(207)
**PTX**	Engineering Microglia and Nanotechnology	Microglia	Microglia were designed as vectors for PTX-targeted delivery using TNTs properties.	(208)

EV, extracellular vesicles; STAT3, signal transducer and activator of transcription 3; CSF-1R, macrophage colony-stimulating factor 1 receptor; PD-1/PD-L1, programmed death-1 and programmed death ligand-1; p38 MAPK, p38 mitogen-activated protein kinase; PVSRIPO, the recombinant oncolytic poliovirus; CL, clodronate liposomes; CTX, cyclophosphamide; OV, oncolytic virus; PSPD3R, purple sweet potato delphinidin-3-rutin; ADI-PEG20, arginine-depleting agents, one kind of arginine deiminases; rAAV2-IL-15, recombinant adeno-associated virus serotype rAAV2 carrying IL-15; CAR-MΦs, GSC-specific chimeric antigen receptor microglia/macrophages; PTX, paclitaxel.

### 4.1 Programmed death-1 and programmed death ligand-1

PD-1, also known as CD279, is an immunosuppressive mediator found in various tumors, gliomas included. PD-L1 is also present in gliomas and correlates with tumor grade (209). Anti-PD-1 therapy in conjunction with radiotherapy has been shown to be effective against glioma in a mouse model (210). Nivolumab is an FDA-approved human IgG4 anti-PD-1 monoclonal antibody that, in conjunction with ipilimumab, is used to treat metastatic melanoma tumors. The effectiveness of this combination therapy for recurrent glioblastoma was assessed in a phase III clinical trial (NCT02017717), but the results suggested that the combination of the two drugs did not confer any survival benefit in terms of overall survival (211). Then for Nivolumab monotherapy, there are also large phase III clinical studies underway (NCT02667587, NCT02617589) for the evaluation of Nivolumab combined with radiotherapy ± TMZ for the management of newly diagnosed glioblastoma patients, where NCT02667587 showed no improvement in patient survival with this regimen (212). One of the contributing factors to the lack of clinical efficacy of this combination of standard RT + anti-PD-1 may be the existence of microglia within the tumor (193). The M1 microglial phenotype contributes to the effectiveness of anti-PD-1 immunotherapy in gliomas, while recruitment and clustering of the M2 phenotype are linked to anti-PD-1 resistance. Additionally, the upregulation of PD-L1 expression in microglia impacts treatment efficacy (213). Immune checkpoint inhibition (ICI) is generally ineffective against glioblastoma, primarily due to its immunosuppressive tumor microenvironment and limited CD8^+^ T cell infiltration. Furthermore, microglia can inhibit CD8^+^ T cell cytotoxicity through several mechanisms during tumor development and treatment. Anti-PD-1 can suppress microglia proliferation and induce apoptosis via antibody-dependent cytotoxicity (ADCC). Moreover, anti-PD-1 antibodies can directly penetrate the glioma microenvironment, kill PD-1-expressing microglia, and convert them to an anti-tumor phenotype. Therefore, in gliomas with limited T-cell infiltration, anti-PD-1 therapy may rely on the substitution of T cells with other immune cells, such as microglia, to exert its anti-tumor effects (214, 215). Thus, removal of microglia from gliomas becomes a possibility to improve the anti-PD-1 effect, and the use of a highly selective CSF-1R inhibitor (PLX5622) to remove microglia across the BBB was proposed by Clausi MG et al. (193). Simultaneous treatment with p38 MAPK inhibitors and PD-L1 antibodies effectively reduced PD-L1 levels in microglia residing within glioma microenvironments, leading to a significant decrease in tumor cell proliferation. This combination therapy significantly extended the survival of patients with temozolomide-resistant glioblastoma (194).

### 4.2 Oncolytic virus

All viruses have a specific cellular orientation, and oncolytic viruses are anti-cancer viruses that have a specific orientation towards tumor cells and therefore do not cause damage to normal cells. Oncolytic viruses kill infected cancer cells through various pathways including direct or indirect virus-mediated cytotoxic immune effect mechanisms and regulation of apoptosis, necrosis, and autophagy of tumor cells. In addition, it can also kill uninfected cancer cells indirectly by directly destroying tumor blood vessels (216). Talimogene laherparepvec (T-VEC), a genetically modified herpes simplex virus (HSV), received FDA approval in 2015 as a therapeutic option for patients with metastatic melanoma (217). In addition to this, several other oncolytic viruses, including adenovirus, retroviruses, measles virus, and poliovirus have all demonstrated some antitumor activity in cancer patients (218). DNX-2401 (Delta-24-RGD/tasadenoturev) is an oncolytic adenovirus designed for targeting tumors that is selective for tumor cells and easy to manipulate and has shown significant effects in both murine models and a phase I clinical trial (219).The recombinant oncolytic poliovirus (PVSRIPO) targets the poliovirus receptor CD155, which is frequently overexpressed in tumors and also shows good results after intra-glioma injection (220). In addition, clinical trials investigating oncolytic viruses based on the HSV strain for glioma treatment are currently underway (NCT00028158, NCT03911388, NCT02062827) (221). The efficacy of OV may be influenced by increased microglia aggregation and infiltration in the tumor. This is because microglia can compete with glioma cells for viral uptake both through direct uptake of the virus by endocytosis and the antiviral response generated by the M1 polarization state. In addition, silencing of the OV gene can also be mediated by the STAT pathway, ultimately leading to a decrease in OV titers and inhibition of subsequent replication (222). A recent investigation has additionally verified that a rise in proliferation and infiltration of microglia around the tumor was indeed monitored during PVSRIPO treatment, and suggested that this antiviral inflammatory response may result from PD-L1 immune checkpoint activation on microglia. Therefore, Yang et al. proposed that the combination of PVSRIPO with PD-1/PD-L1 blockade could potentially extend the transient antitumor effect (196). The use of clodronate liposomes (CL) as well as cyclophosphamide depletes tumor microglia and inhibits the release of their antiviral factors to increase OV titers and gene expression, ultimately improving efficacy (197, 198). At the same time, several experiments targeting inhibition of microglia activity have shown a facilitative effect on OV therapy. For example, by using the immunosuppressive factor TGF-β to temporarily suppress tumor microglia and thus enhance the efficacy of OV (223).

### 4.3 Potential drug

#### 4.3.1 CSF-1R inhibitor

Microglia can be invaded by glioma cells mediated by CSF-1R signaling on gliomas, a pathway involving CSF-1 signaling emitted by ERK(extracellular regulated protein kinases) (224). In a murine model, treatment with the CSF-1R inhibitor (PLX3397) resulted in decreased microglia activation and proliferation to prevent glioma cells from invading the brain parenchyma (80). However, PLX3397 was found to hinder the differentiation of monocytes into macrophages, rather than inhibiting monocyte influx into the tumor (28). In the clinical trial (NCT01349036), the drug exhibited effective BBB penetration and acceptable patient tolerability, the preliminary findings of the trial did not demonstrate significant antitumor effects of the inhibitor (225). A phase II clinical trial (NCT01790503) investigated the efficacy of combining PLX3397 with TMZ and radiotherapy, preliminary results of the trial also showed no significant clinical effect (226). Another CSF-1R inhibitor (BLZ945) was also found to impede tumor growth in a murine model. GM-CSF and IFN-γ, secreted by gliomas, sustained the viability of microglia in the presence of CSF-1R inhibition (195). Although CSF-1R inhibitors have shown prolonged overall survival in glioma models, they are also associated with a certain percentage of recurrences. At the same time, the poor effect shown during human clinical trials may be associated with acquired resistance, which may be related to the aforementioned increase in tumor-secreted GM-CSF and IFN-γ and the increased expression of immune checkpoint molecules, including PD-L1. Elevated activation of the phosphatidylinositol 3-kinase (PI3K) pathway, which is instigated by insulin-like growth factor-1 (IGF-1) and the insulin-like growth factor-1 receptor (IGF-1R) on tumor cells, is detectable in recurrent tumors (227, 228).

#### 4.3.2 Flavonoids

Natural flavonoids and synthetic derivatives have been previously shown to exhibit clear antitumor activity in malignant tumors such as breast cancer and melanoma (229). A 2017 study by Trevor A Stump et al. proposed that apigenin also has antitumor properties and the ability to promote apoptosis in glioblastoma (230). Flavonoids are generally classified into seven types, of which Apigenin, Rutin, and Quercetin represent flavonoids that can traverse the BBB effectively and impede the progression of glioma via modulation of distinct signaling pathways (231). Apigenin directly affects glioma cell migration by modulating IL-6 levels and also induces a reversal from the M2 polarized phenotype to the M1 phenotype of microglia and enrichment towards the tumor region. This effect is dose-dependent and remains non-toxic in an anti-tumor context (199). Rutin and Quercetin, which modulate the chemotaxis of glioma cells to microglia and induce microglia proliferation and restore their original immune response to inflammation, and modulate HDGF, IGF and GDNF levels to impede the growth and invasiveness of glioma cells (200). Wang M’s team extracted Rutin from purple potato and named purple sweet potato delphinidin-3-rutin (PSPD3R) which, additionally possessing the above-mentioned functions, was found to induce autophagy and apoptosis in glioma cells to achieve inhibition of glioma proliferation (201).

#### 4.3.3 Arginine-depleting agents

Tumor cells exhibit a heightened requirement for arginine, a semi-essential amino acid that is synthesized via the participation of argininosuccinate synthase (ASS) (232). Targeted arginine-depleting antitumor approaches become a potential possibility, and arginine-depleting agents (arginine deiminase ADI-PEG20) not only markedly suppressed tumor growth in a mouse model of glioma, but also acted synergistically with TMZ to enhance its effect in glioma (202). In addition, ADI-PEG20 augmented the radiosensitivity of glioma cells that are positive for argininosuccinate synthase 1 (ASS1) to ionizing radiation (IR) (i.e., sensitivity to radiotherapy) and also promoted further infiltration of microglia into the tumor, shifting their pro-tumor phenotype to an anti-tumor phenotype and exerting anti-tumor effects (203).

### 4.4 Engineering microglia and nanotechnology

Zoledronate (ZOL)-loaded microglia membrane nanoparticles (ZOL@CNPs), which can target drug ZOL delivery to tumor regions by exploiting the recruitment mechanism of tumor cells to microglia, can inhibit tumor cell proliferation and invasion by increasing M1 phenotype microglia and blocking HIF-1α expression, ultimately achieving TMZ-resistant glioma progression inhibition (233). Polyamindoamine (PAMAM) can effectively cross the BBB and tumor barrier, and surface modification with sugar molecules will enable the dendrimer polymer to expand its microglial cell targeting promising as a targeting vehicle for antitumor drugs. In addition, AD, an amphiphilic PAMAM dendrimer, can complex with siRNA to form stable nanoscale particles, thereby preventing its degradation, reduce its target gene and protein expression after being taken up by microglia. Since this approach does not affect the other normal functions of microglia, it can be expected to be a harmless carrier for siRNA delivery to microglia (234, 235). miRNAs are key regulators of microglia polarization, with miRNA-155 having prominent significance for M1 polarization. Gao X’s team developed a membrane-encapsulated nucleic acid nanogel, Vir-Gel, embedded with a therapeutic miR-155-like virus, which can reprogram microglia from M2 phenotype to M1 phenotype to achieve tumor suppression (204). In addition, either intranasal delivery of a siRNA CD73-loaded cationic nanoemulsion or rAAV2-IL-15 (recombinant adeno-associated virus serotype rAAV2 carrying IL-15) modified microglia, or injection of a hydrogel containing the CXCL12 blocker Plerixafor (AMD3100) into the surgical cavity, can improve the immunosuppressed tumor microenvironment by blocking microglia recruitment and infiltration, thereby inhibiting tumor progression or recurrence (205, 206, 236). Chen C et al. reported an injectable nanocarrier-hydrogel superstructure into the postoperative cavity in which the nanocarrier introduces GSC-targeted CAR genes into the nucleus of microglia, thereby generating GSC-specific chimeric antigen receptor microglia/macrophages (CAR-MΦs) in the surgical cavity to remove residual GSCs and prevent postoperative glioma recurrence (207). Microglia can engage in cell-to-cell communication with numerous cell types in the brain, such as glioma cells, via tunneling nanotubes (TNTs) (237). Using the properties of tunneling nanotubes (TNTs), microglia can be engineered to enhance the targeted delivery of the anticancer drug paclitaxel (PTX). Loading PTX with liposomes (PTX-Lp) and engulfment by microglia circumvents the non-selective cytotoxicity of PTX and gives it the ability to efficiently transmigrate through the BBB (208). Guo L’s team proposed that iron oxide-nanoparticles and the near-infrared fluorescent dye DiD could be used to engineer microglia BV2 as an intraoperative optical imaging agent carrier (DiDBV2-Fe), which could be considered for intraoperative display of tumor boundaries and guidance of surgery given its efficient transmission through the BBB with no significant adverse effects. It may also be superior to commercially available 5-ALA, but there remains a potential clinical immune rejection issue (238).

## 5 Discussion

As a serious primary brain tumor plaguing humans, glioma has a high morbidity and mortality rate, usually with a strong local invasive capacity and a poor overall prognosis for patients. Early investigators believed that this particular physiological structure of the BBB gave the brain its so-called immune privilege, and now, as related studies continue, the significance of TAMs in the TME is increasingly being recognized. An increasing body of ex vivo evidence is also corroborating that it is the tight interplay between TAMs and various cells in the TME that contributes to the distinctive immune-suppressive properties of gliomas. Microglia and BMDMs are the main cellular components that constitute TAMs, and their crosstalk with gliomas deserves further exploration. At this moment, it is challenging to differentiate between BMDMs and microglia in tumors either by cell morphology or by the surface specific antigen CD45, and then it is even more difficult to understand the specific functional roles of the two cell subpopulations. Therefore, in order to facilitate the study of the interactions between microglia and gliomas, we should clarify the heterogeneity of these two cell subpopulations. The heterogeneity between microglia and BMDMs is mainly reflected in the cytological origin, the different temporal and spatial distribution in the tumor, and the specific markers. Although a few microglia are replaced in gliomas with damaged BBB, immature yolk sac progenitor cells are now considered to be the primary microglia initiating cells, which differ from the bone marrow origin of BMDMs. In general, microglia tend to accumulate in the marginal regions of tumors, whereas BMDMs are primarily enriched within tumors, but this distribution changes with tumor progression and recurrence or even tumor type. Both the traditional differentiation method based on the macrophage surface antigen CD45 and the later microglia marker CX3CR1 are deficient because they are expressed in both cell subpopulations and therefore are not specific enough. The current study proposes that TMEM119 and P2RY12 can be considered as markers of microglia while CD49D and CD163 can be considered as markers of BMDMs.

The presence of microglia substantially augments the proliferative and migratory capabilities of glioma cells, suggesting that this may be due to the active mediators secreted by microglia. For example, CSF family, interleukin family, TGF-β, etc. At the same time, EVs sreleased by microglia and glioma cells, along with the activation of relevant surface receptors on both cell types, are pivotal in mediating the crosstalk process. Since the BBB is often disrupted in the context of malignant glioma and its permeability is increased, exogenous macrophages (BMDMs) tend to pass through the BBB to tumor-associated areas and gradually accumulate as a major infiltrating cell subpopulation. Concurrently, tumor-secreted chemokines recruit resident microglia in the brain, which undergo a transformation into a macrophage-like immune phenotype upon activation of multiple signaling pathways, including STAT3, NF-κB, mTOR, PI3K/Akt, and Wnt/β-catenin. This immune phenotype of microglia contributes to tumor progression by exerting an immunosuppressive effect.

Even with timely surgical resection of the lesion and standard postoperative treatment protocols, patients with glioma still face serious postoperative complications, highlighted by neurological impairment, which can significantly reduce the quality of survival. In particular, grade IV glioblastoma is not only prone to recurrence due to its high malignancy, but may also be resistant to conventional radiotherapy treatment. In addition, HGG such as glioblastoma is also less responsive to immunotherapy such as ICB, which has become popular in recent years. Existing evidence indicates that activated microglia in the tumor microenvironment can inhibit the activity, function, and quantity of tumor-infiltrating lymphocytes (TILs, like CD4+ or CD8+ T cells) with anti-tumor effects. This exacerbates the immune escape of glioma cells and their tolerance to common immunotherapies (such as Anti-PD-1 therapy), and may even eventually lead to patient relapse. However, current research is largely limited to the macroscopic mechanisms between common effector lymphocytes and microglia. The microscopic interaction between the two is not yet well understood, and no specific mechanisms of interaction have been clearly identified to provide new solutions for gliomas. In addition, the interaction between potential tumor-killing cells other than common T cells (B lymphocytes, natural killer cells, dendritic cells, polymorphonuclear neutrophils, γδ T cells, etc.) and microglia remains unclear and requires further research. In particular, it is worth exploring whether microglia will have similar inhibitory effects on these cells, thereby promoting tumor progression. Of course, combination of the relatively mature PD-1/PD-L1 immunotherapy with other treatments has shown promise in improving clinical outcomes. OV treatment regimens, engineered microglia and some potential agents (CSF-1R inhibitor, Flavonoids, Arginine-depleting agents) can also be considered as a new attempt to treat glioma. Based on such findings, then, it is reasonable to hypothesize that the combination of the aforementioned emerging treatments may produce more significant clinical results. However, this also requires more experiments in the future to verify this idea. Therefore, we can reasonably assume that the combination of multiple effective treatments should become a maturing trend in the future.

Whether it is the inability of most experimental drugs to reproduce their effectiveness when entering clinical trials or the lack of analysis of crosstalk studies between microglia and gliomas, We can find that this is due to the fact that there is still a lack of a reliable *in vivo* and ex vivo experimental model. Because murine models are still the main platform for research and experimentation, mouse and human microglia tend to have different altered gene expression profiles in the glioma setting and the source of cells is often limited to intraoperative collections (which is the period when microglia exert their strongest immunosuppressive effects), which means that the already complex immune microenvironment of the human brain cannot be perfectly reproduced (239, 240). Several teams have made new attempts, simple tissue engineered hydrogel platform models, peripheral blood-derived microglia-like (iMG) cells can serve as a predictor for the microglia in tumors and Microfluidic cell culture assays with full phase detection capability have contributed to the evolution of the experimental model (240–242).

## Author contributions

Study conception and design: NL, J-CT, DY. Literature search and review: WS, D-RZ, YW, S-QH, KD. Manuscript writing and revision: NL, J-CT, DY. All authors contributed to the article and approved the submitted version. J-CT and DY contributed equally to this work and share first authorship.
